# Endovascular thrombectomy versus medical treatment for large vessel occlusion stroke with mild symptoms: A meta-analysis

**DOI:** 10.1371/journal.pone.0203066

**Published:** 2018-08-23

**Authors:** Yong-Jie Xiong, Jia-Ming Gong, Yi-Chi Zhang, Xin-ling Zhao, Sha-Bei Xu, Deng-Ji Pan, Wen-Sheng Qu, Dai-Shi Tian

**Affiliations:** 1 Department of Neurology, Tongji Hospital, Tongji Medical College, Huazhong University of Science and Technology, Wuhan, P.R. China; 2 Department of Neurology, Central Hospital of Ankang City, Shanxi, P.R. China; Universitat Regensburg, GERMANY

## Abstract

It remains controversial as to whether mechanical thrombectomy (MT) is safer and more beneficial in patients with large vessel occlusion stroke (LVOS) presenting with a National Institutes of Health Stroke Scale score ≤ 8. We therefore conducted a meta-analysis of the published data.We searched PubMed and Embase and pooled relevant data in the meta-analyses using fixed effects models. Only studies that directly compared best medical therapy alone (BMT) with MT were included. We used odds ratios to analyze the associations between MT and 90-day functional outcome (evaluated using the modified Rankin Scale (mRS)), mortality, and rates of symptomatic intracerebral hemorrhage (sICH) in patients with LVOS and minor symptoms. Five studies including a total of 581 patients met our inclusion criteria. A significant difference was found that the patients treated with MT were associated with improved 90-day mRS score (OR, 1.68; 95% CI, 1.08–2.61) compared with BMT group. There was no difference in 90-day mortality between the two groups. However, sICH occurred more frequently in the MT group than the BMT group (OR, 3.89; 95% CI, 1.83–8.27). Patients with LVOS with minor or mild symptoms who underwent primary thrombectomy had a significantly improved 90-day mRS score compared to those who received BMT alone. Meanwhile, the risk of sICH was higher in the MT group than that in BMT group. Future randomized clinical controlled trials evaluating the role of endovascular reperfusion for LVOS with minimal symptoms are warranted.

## Introduction

According to the guideline of ischemic stroke outlined in 2015[1], mechanical thrombectomy (MT) is considered to be the standard treatment for large vessel occlusion stroke (LVOS) of the anterior cerebral circulation and benefits the patients presenting with National Institutes of Health Stroke Scale (NIHSS) score ≥ 6 within 6 hours. Most clinical trials of MT have excluded patients with minor to mild stroke and with NIHSS scores of six to eight or lower, except for a few patients of the Multicenter Randomized Clinical Trial of Endovascular Treatment for Acute Ischemic Stroke in the Netherlands (MR CLEAN) and the Extending the Time for Thrombolysis in Emergency Neurological Deficits—Intra-Arterial (EXTEND-IA)[2]. For predicting LVOS, a prospective study showed only a 48% sensitivity of higher NIHSS scores[3]. However, a significant proportion of patients presenting with LVOS and minor symptoms subsequently decline and end up with poor clinical outcomes, and it remains unknown as to whether those patients with LVOS and minor symptoms benefit from MT. Considering the low potential recanalization rate with intravenous tissue plasminogen activator (tPA) alone and the poor clinical outcomes reported at 90 days[4], a more positive recanalization therapy such as MT may be more beneficial. Recently, some clinical trials have compared the efficacy and safety between MT and the best medical treatment (BMT) in patients with LVOS with mild symptoms; however, their results are divergent. Thus, we conducted a meta-analysis of published studies to determine whether MT is safer and more beneficial than BMT for patients with LVOS presenting with NIHSS scores ≤ 8.

## Methods

### Search strategy and selection criteria

The article was prepared with reference to the Preferred Reporting Items for Systematic Reviews and Meta-Analyses (PRISMA) [5]. The core question this paper tried to answer can be illustrated as a common framework PICO (Patient Population, Intervention, Control, Outcome), as follows: Did patients with LVOS with an NIHSS score ≤ 8 (patient population) and who underwent MT (intervention) have better functional outcomes and lower rates of mortality and symptomatic intracerebral hemorrhage (sICH; outcomes) than patients who received BMT alone including thrombolysis(control)? We searched Pubmed and Embase using the following search terms: (mild stroke OR minimal stroke OR minor stroke) AND (thrombectomy OR mechanical thrombectomy OR stent retrievers OR endovascular therapy OR embolectomy OR "thrombectomy"[Mesh]). The resulting references were embedded into the reference manager EndNote X7 (Thompson Reuters, Philadelphia, PA) and duplicate references were removed. Case reports and conference abstracts were also excluded. Two authors screened the titles and abstracts of journal articles systematically and independently, and any uncertainties were discussed before the final inclusion of studies was decided.

Studies were eligible for inclusion if they had (1) defined large vessel occlusion by radiology image, (2) included patients with an NIHSS score ≤ 8 at the onset, and (3) assessed the rate of sICH and 3-month functional outcome after MT or BMT. Studies were excluded if they included pediatric patients or had used intra-arterial pharmacotherapy during intervention.

### Quality assessment and data extraction

Finally, five studies[6–10] were included after a full-text reading of articles. We used the Newcastle-Ottawa scale to evaluate the methodological quality of observational trials and cohort studies, while 6 asterisk or more stars indicated high quality. From each included study, data from patients with the following outcomes in the MT and BMT groups were extracted independently by two authors: 90-day modified Rankin Scale (Mrs) score of 0 to 2, death within 90 days, and occurrence of Sich.

### Meta-analyses

Using the version 5.3 of Review Manager (RevMan) software, dichotomous data from searched studies were extracted to generate odds ratios (Ors) with 95% confidence intervals (Cis), and heterogeneity across studies was tested using the I^2^ statistic. To calculate a summary OR with 95% Cis, a meta-analysis was performed using a Mantel–Haenszel fixed effects model when the I^2^ statistic was below 50%. Furthermore, we conducted sensitivity analyses to explore possible explanations for heterogeneity by omitting each study in turn. We meant to evaluate the publication bias and related biases through funnel plots, but the number of included studies was too small to make much sense. Finally, we performed Egger test to evaluate the publication bias, and P < 0.1 regarded as significant asymmetry by the means of STATA software version 12.0.

## Results

### Study characteristics

**Fig 1:**
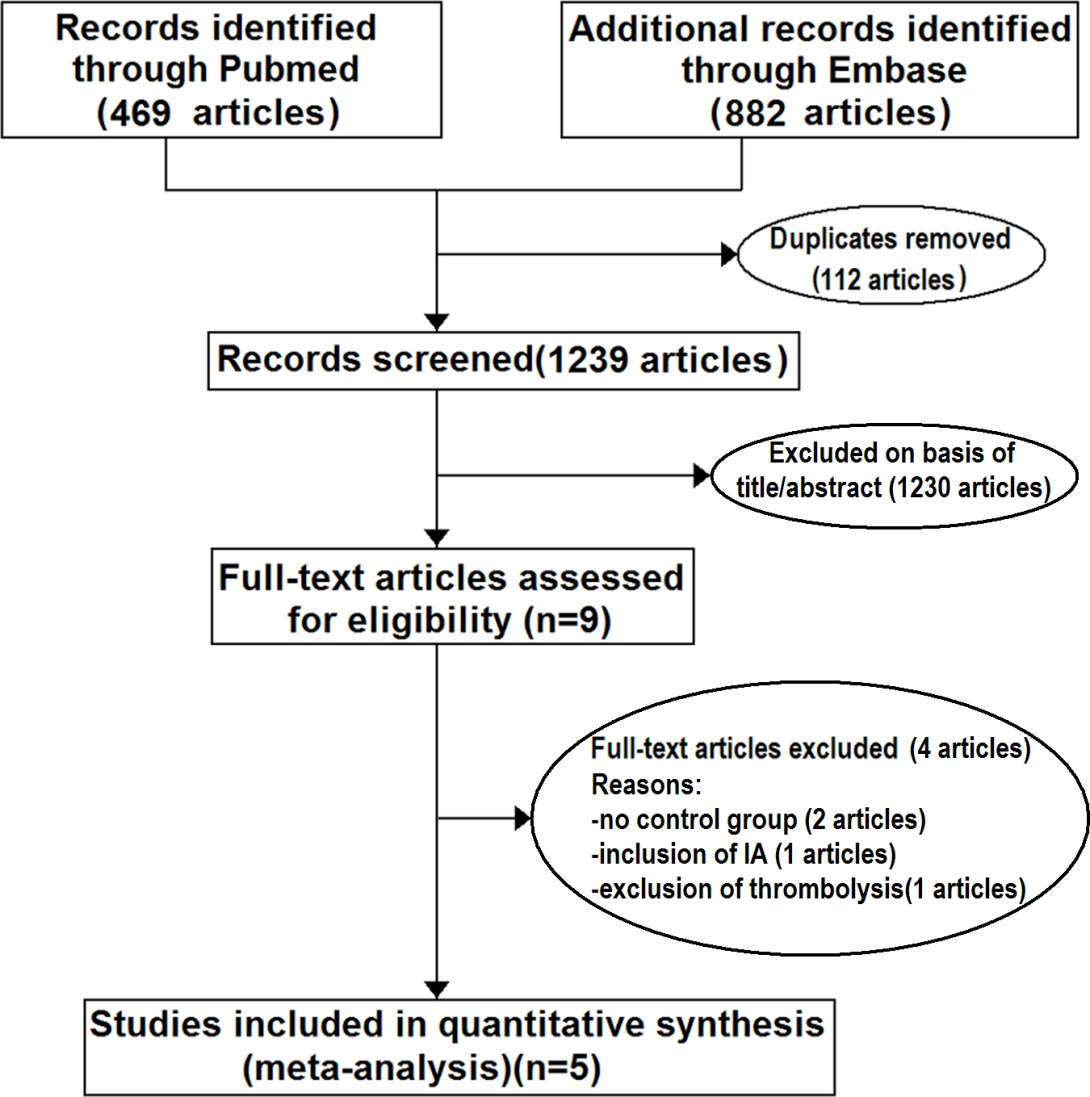
Flow chart of literature search and study selection.

According to above search strategy, we acquired 1351 articles from Pubmed (from January 1, 1993 to December 31, 2017) and Embase (from January 1, 2009 to December 31, 2017). After duplicates had been removed and abstracts screened, nine studies were left. Through comprehensive reading, four studies were excluded due to: (1)an absence of a control group[11–12], (2)more other endovascular treatments than MT and unobtainable detailed data from authors[13], and (3)the purposeful exclusion of thrombolysis from medical treatment[14] (Fig 1). Five studies[6–10] including a total of 581 patients met our inclusion criteria. Among these, 250 patients who underwent MT (MT group) and 331 patients who received BMT, including thrombolysis (BMT group), were pooled for the meta-analysis. The scores of Newcastle-Ottawa scale of included studies ranged from 6 to 9 stars. The characteristics of the included studies are presented in Table 1. In the BMT group, 39 patients showed clinical deterioration and received MT treatment in consideration of ethics[7,9–10].

**Table 1:** Characteristics of the patients in the included studies.

	X.Urra[6]		DC Haussen 2016[7]		DC Haussen 2017[8]		MP Messer[9]		C Dargazanli[10]	
	MT group (n = 34)	BMT group (n = 44)	MT group (n = 10)	BMT group (n = 22)	MT group (n = 30)	BMT group (n = 88)	MT group (n = 8)	BMT group (n = 46)	MT group (n = 170)	BMT group (n = 131)
Age	64±14.8	71±12.5	60±13.9	68.2±14.4	63.5(56.5–72.5)	73(59–80.7)	63(32–78)	71(40–95)	63.6±15.7	69.4±15.4
Sex(male)	24	26	5	15	15	41	3	27	95	65
Hypertension	21	29	6	13	21	63	2	32	80	69
Diabetes	6	6	2	4	9	10	2	9	19	15
Dyslipidemia	14	22	2	5	9	29	1	21	39	60
Atrial fibrillation	9	14	3	8	10	16	2	16	67	46
OTT(min)	140 (69–230)	132.5 (66–181)	375 (225–547.2)	630 (258–900)	300 (174–498)	294 (120–1002)	214±150	213±145	128 (94–184)	169 (122–282)
Occlusion site										
ACA	0	0	1	0	1	0	0	0	0	0
MCA M1	13	13	6	10	11	17	6	12	92	57
MCA M2	7	16	1	5	9	43	0	22	44	47
intracranial ICA	0	1	0	3	2	17	2	12	10	5
extracranial ICA	0	0	0	1	0	0	0	0	9	6
tandem	2	2	0	3	0	0	0	0	15	16
basilar	12	12	2	3	7	11	0	0	0	0
Intravenous Tpa	16	29	6	2	12	8	5	46	103	53
baseline NIHSS	4(3–5)	3(3–5)	4(2–5)	2(1–4)	1(0–2)	1(0–3)	2(0–5)	4(2–5)	5(3–6)	3(2–6)
NOS	*******		******		*******		******		*********	

MT: mechanical thrombectomy; BMT: best medical treatment; OTT: onset to treatment; ACA: anterior communicating artery; MCA: middle cerebral artery; ICA: internal carotid artery; tPA: tissue plasminogen activator; NIHSS: National Institute of Health Stroke Scale; NOS: Newcastle-Ottawa scale, observational studies achieving 6 asterisk (*) or more were considered to be of high quality.

According to the data provided by Urra[6] via E-Mail, two patients who received intra-arterial pharmacological treatment in the endovascular treatment group were excluded and the remaining 32 patients were included in the MT group of our meta-analysis. Unfortunately, the author directly classified the deteriorated patients into the endovascular treatment group and could not offer the detailed characteristics of them, so we had to enroll the data as verbatim from the original paper (there will be a separate analysis in the latter). The study by Haussen et al[8] did not describe the above situation. All studies considered a mRS score of 0 to 2 to indicate a good or favorable functional outcome, and defined an excellent outcome as an mRS score of 0 to 1. A sICH was defined as either a ≥ 4-point increase in the NIHSS score or as hemorrhagic transformation graded by the European Cooperative Acute Stroke Study criteria.

### Functional outcomes

**Fig 2:**
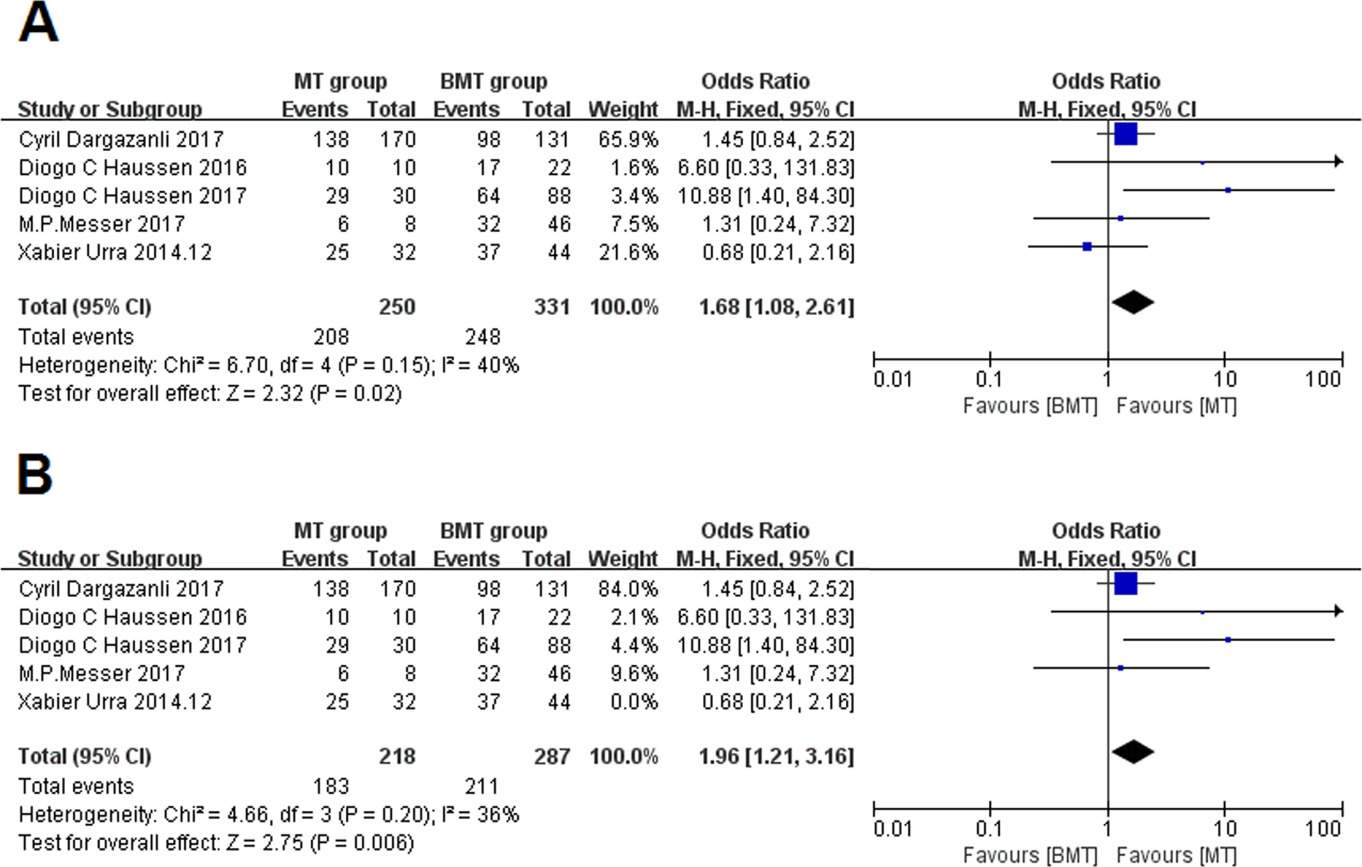
Forest plots of good outcomes. **A:** Forest plots showing the odds ratios of good outcomes (90-day mRS of 0–2) in patients treated with mechanical thrombectomy (MT) vs. those treated with best medical treatment (BMT). **B:** Forest plots showing the odds ratios of good outcomes (90-day mRS of 0–2) in patients treated with MT vs. those treated with BMT excluding the study by Urra X et al.

The primary outcome of the present review was the 90-day mRS score of 0 to 2 for functional outcome. The meta-analysis demonstrated that the pooled proportion of patients in the MT group with an mRS of 0 to 2 was 83.2%, vs. 74.9% in the BMT group, and the OR is 1.68 (95% CI: 1.08–2.61, I^2^ = 40%). A significant difference was found between MT and BMT groups for the proportion of patients with mRS 0 to 2, which indicated a more favorable functional outcome in the MT group (P = .02)(Fig 2A). Moreover, the OR (1.96; 95% CI, 1.21–3.16) of favorable outcomes further increased when the study by Urra et al[6] was excluded from the analysis (Fig 2B). No publication bias were found. (Egger's test P = .69 and .53 respectively).

**Fig 3:**
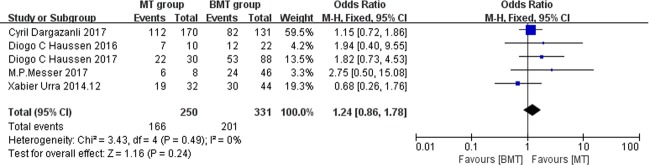
Forest plots of excellent outcomes. Forest plots showing the odds ratios of excellent outcomes (90-day mRS of 0–1) in the patients treated with MT vs. those treated with BMT.

The secondary outcome was the 90-day mRS score of 0 to 1, which indicates excellent outcome. The pooled proportion of patients in the MT group who had an mRS score of 0 to 1 was 66.4%, and this was 60.7% in the BMT group, the OR is 1.24 (95% CI: 0.86–1.78, I^2^ = 0%); however, there was no significant between-group difference in the proportion of patients with an mRS score of 0 to 1 (P = .24; Fig 3). No publication bias were found. (Egger's test P = .43)

### Mortality

**Fig 4:**
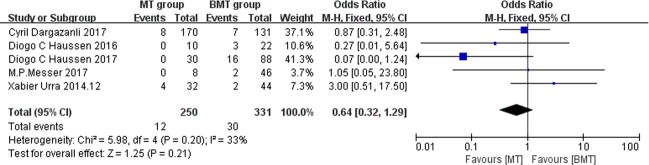
Forest plots of mortality. Forest plots showing the odds ratios of mortality in the patients treated with MT vs. those treated with BMT.

The pooled mortality rate was 4.8% in the MT group and 9.1% in the BMT group, the OR is 0.64 (95% CI: 0.32–1.29, I^2^ = 33%), but there was no significant difference detected (P = .21; Fig 4).

### Symptomatic intracerebral hemorrhage

**Fig 5:**
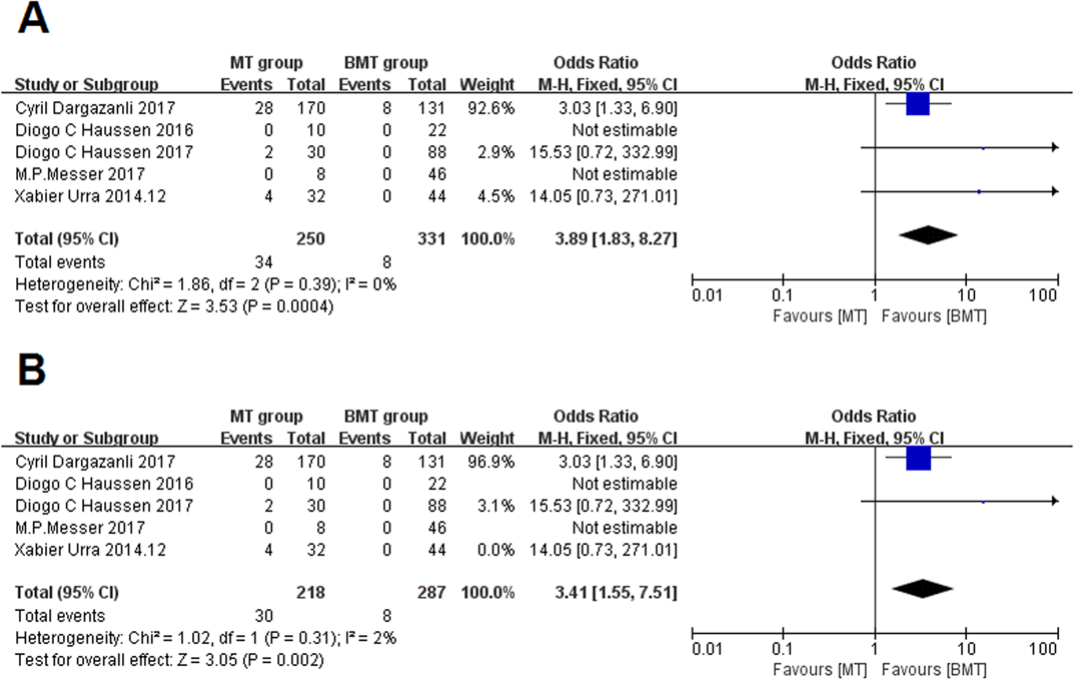
Forest plots of symptomatic intracerebral hemorrhage. **A:** Forest plots showing the odds ratios of symptomatic intracerebral hemorrhage in the patients treated with MT vs. those treated with BMT. **B:** Forest plots showing the odds ratios of symptomatic intracerebral hemorrhage between the patients treated with MT and those treated with BMT excluding the study by Urra X et al.

The proportion of patients with sICH was 13.6% in the MT group and 2.4% in the BMT group, the OR is 3.89 (95% CI: 1.83–8.27, I^2^ = 0%), and this difference was significant (P = .0004; Fig 5A). When the study by Urra[6] was excluded, the proportion of sICH incidence in the MT group decreased; however, the tendency of the difference between MT and BMT groups persisted (OR, 3.41; 95% CI, 1.55–7.51) (Fig 5B).

## Discussion

We performed a meta-analysis on outcome data reported in the literature of patients with LVOS with minor symptoms undergoing MT or not. Our aim was to understand whether MT provided additional benefits for patients with LVOS and a low NIHSS score compared with BMT alone. Our results indicated that primary thrombectomy significantly improved 90-day mRS scores compared with BMT alone, but that the risk of sICH was higher.

A long-held belief is that minor or mild stroke is associated with good functional outcomes, whether treated or not. The median NIHSS score for those patients included in the most cited five randomized controlled trials[15–19] comparing MT plus intravenous alteplase with thrombolysis alone was approximately 13 to 18, and thus excluded the vast majority of patients with NIHSS scores of six to eight or lower. However, a low NIHSS score is not always accompanied by good functional outcomes. Nedeltchev et al.[20] demonstrated that 23.5% of 162 patients with acute ischemic stroke and initially mild symptoms experienced poor neurological outcomes, and 1.3% eventually died. A similar report of 378 patients with mild stroke onset found that early neurological deterioration occurred in 14.6% of patients[21]. In fact, a considerable proportion of acute ischemic stroke with low NIHSS scores and defined as minor or mild stroke appear to be due to large vessel occlusion[3], which has been used to predict 6-month mortality (OR, 4.5; 95% CI, 2.7–7.3; P < 0.001) and to negatively predict 6-month good outcomes[22]. These studies indicate that the presence of large vessel occlusions is independently associated with poor outcomes, regardless of symptom severity. Therefore, mild stroke seems not to be a “mild problem”. Some mild stroke patients with thrombolysis alone showed low potential recanalization rate. Hence, more positive recanalization treatment should be performed according to the presence of large vessel occlusions rather than mild symptoms.

Several studies have suggested that MT subsequent to optimal medical therapy might improve the prognosis of patients with LVOS and mild symptoms. In one study, 75% of 41 patients with LVOS and a low NIHSS score (≤ 5) who received MT had a good outcome (an mRS score 0–2) at the 90-day follow-up[23]. In a retrospective analysis of 484 patients with stroke who received MT[12], 33 patients with LVOS and low NIHSS scores (≤ 8) were identified, of which 21 (63.6%) and 30 (90.9%) achieved favorable (mRS score 0–2) and moderate (mRS score 0–3) clinical outcomes at 90 days. Dargazanli et al.[11] analyzed 138 patients with LVOS and with an NIHSS score < 8 who underwent MT and reported that a favorable outcome (an mRS score ≤ 2) was achieved in 108 patients (78.3%) and death occurred in only 7 (5.1%). Thus, the early verification of large vessel occlusion through radiological screening is extremely important and may affect the choice of potential treatment means, including MT.

Moreover, the efficiency and safety of BMT alone (including thrombolysis) vs. additional MT in patients with LVOS with mild symptoms has been compared in several studies[7–10]. However, there is no unified conclusion so far. Hassen et al.[7] prospectively enrolled 32 consecutive patients with NIHSS scores ≤ 5 and large vessel occlusions confirmed by computer tomography angiography in a single-center study; 22 patients were treated with medical therapy and 10 patients were treated with MT. The 90-day mRS 0–2 rates were 77% and 100%, respectively (p = 0.15), and 41% of patients treated with medical therapy had worsening symptoms and required MT for urgent rescue. Another multicenter retrospective study from the same group[8] reported results from 88 patients that received medical management and 30 that received MT. The rate of mRS 0–2 at the 90-day follow-up was 72.7% and 96.7%, respectively (p = 0.01), and that of 26 pairs produced by the matched analysis were 69.2% and 93%, respectively (p = 0.04). Messer et al.[9] prospectively selected 54 (14.2%) patients with minor LVOS and found that the rate of excellent outcome (an mRS score 0–1) was higher in patients with immediate MT (75%, 6/8) compared with medical management only (55%, 22/40), but rates of good outcome (an mRS score 0–2) were similar. When the rest 6 patients with rescue MT were classified into the medical management group, the outcome of the MT group was seemingly better (75% vs. 69.6%). Dargazanli et al.[10] published a multicenter cohort study involving 301 acute ischemic stroke patients withminor and mild symptoms (i.e. with an NIHSS score < 8) harboring large vessel occlusions andtwo therapeutic approaches of urgent MT plus best medical management and best medical management alone. A total of 138 out of 170 patients (81.2%) with MT achieved good outcome (mRS 0–2) at 3 months, and 98 out of 131 patients (74.8%) with best medical management only achieved a good outcome, but no difference was found between the two groups. It is worth noting that the group with best medical management alone also included 24 patients who received MT as rescue therapy because of early neurological worsening. In another observational, prospectively collected, multicenterstudy[6] with 78 consecutive patients within 6 hours of stroke, an NIHSS score ≤ 5 at presentation, and large vessel occlusion similar functional outcomes at 3 months were found for MT and best medical management alone. In recent research, Simon Nagel et al.[24] compared immediate MT (n = 80) with best medical management including rescue MT (n = 220) in patients with acute LVOS and with mild symptoms (NIHSS score ≤ 5), and reported that the OR for a good outcome (mRS 0–2) was 3.1 (95%CI: 1.4–6.9), thus favoring immediate MT and reporting no safety concerns. Amrou Sarraj et al.[25] also found a possible benefit of MT for patients with mild stroke and with NIHSS scores of 4–5 (57.1% MT vs. 22.2% BMT, aOR 4.04, 95% CI: 2.56–6.38, p < 0.01) and no additional benefit with an NIHSS score ≤ 3 (51.9% MT vs. 74.6% BMT, aOR 0.39, 95% CI: 0.25–0.61, p < 0.01). Our meta-analysis based on the above studies found that patients with LVOS and minor or mild symptoms in the MT group had better functional outcomes (90-day mRS scores) and a similar mortality compared to the BMT group. These results indicate that MT should not be forgottenas an optional treatment in patients with LVOS and mild symptoms. Further randomized clinical controlled trials and more detailed grouping of patients with LVOS according to NIHSS scores may be needed to confirm these results.

Despite the potential benefits of MT for patients with mild stroke due to large vessel occlusion, the risk of sICH was higher in the MT group in most of included references[6,8,10]. Hassen et al.[7] and Messer et al.[9] found no patients with sICH in the MT group, but their sample sizes were relatively small. Further research is needed to explore the cause of sICH after MT so that patients can be selected more appropriately. In the included studies, most patients who initially received medical therapy subsequent to rescue MT when deteriorated were grouped into the BMT group; this was not true for the study of Urra et al.[6], which classified such patients into the MT group, details for which could not be extracted from the data. After excluding this study from the analysis, the OR of patients with LVOS with good outcomes increased and sICH decreased, which suggests that rescue MT may be associated with bad outcomes and sICH. In summary, it may be necessary to classify deteriorated patients with rescue MT into a separate subgroup in future clinical studies.

Our results must also be interpreted with consideration to the limitations of this study. First, recanalization rates were not evaluated in this article because of the absence of related information in several included studies. However, the relationship between the outcome and recanalization rate is not actually clear. Treatment with intravenous thrombolysis alone in LOVS is associated with a low recanalization rate[26], which may be explain the worse outcome of LOVS with medical treatment alone. Vascular recanalization is usually considered as successful MT treatment and has been associated with good outcomes, but cerebral hyperperfusion may lead to different outcomes[27–28]. Second, patients who underwent MT in these studies[7–8] received more intravenous thrombolysis than controls, which may lead to better outcomes in the MT group, but also more sICHs. Third, the potential heterogeneity of symptom onset to treatment may have affected the results. Besides, the sample size (n = 581) was not large enough and better designed comparative studies in patients with LOVS should be performed to reveal the effect of MT when the symptoms are minor or mild.

## Conclusions

Mild stroke is actually not a “mild problem”, since low NIHSS scores do not always equate to good functional outcomes, especially in the case of LVOS. It remains elusive as to whether patients with large vessel occlusion and minor symptoms benefit from primary thrombectomy techniques. Our meta-analysis demonstrated that patients with LVOS and minor/mild symptoms (NIHSS score < 8) that underwent MT achieved better functional outcomes at 3 months compared to those administrated with BMT, although these patients also had a higher rate of sICH. However, differences in baseline characteristics might explain these results, and randomized clinical controlled trials evaluating the role of endovascular reperfusion for LVOS with minimal symptoms are therefore warranted.

## Supporting information

S1 File PRISMA checklist. (DOC)
